# Poly(Ionic Liquid): A New Phase in a Thermoregulated Phase Separated Catalysis and Catalyst Recycling System of Transition Metal-Mediated ATRP

**DOI:** 10.3390/polym10040347

**Published:** 2018-03-21

**Authors:** Lan Yao, Bingjie Zhang, Hongjuan Jiang, Lifen Zhang, Xiulin Zhu

**Affiliations:** 1 Suzhou Key Laboratory of Macromolecular Design and Precision Synthesis, Jiangsu Key Laboratory of Advanced Functional Polymer Design and Application, State and Local Joint Engineering Laboratory for Novel Functional Polymeric Materials, Department of Polymer Science and Engineering, College of Chemistry, Chemical Engineering and Materials Science, Soochow University, 199 Ren-ai Road, Suzhou 215123, China; 20174209022@stu.suda.edu.cn (L.Y.); zbj593971779@163.com (B.Z.); xlzhu@suda.edu.cn (X.Z.); 2 Changzhou Huake Polymers Co., Ltd., 602 Yulong Road, Xinbei District, Changzhou 213022, China; 3 Global Institute of Soft Technology, No. 5 Qingshan Road, Suzhou National Hi-Tech District, Suzhou 215163, China

**Keywords:** poly(ionic liquid)s (PILs), atom transfer radical polymerization (ATRP), thermoregulated phase separated catalysis (TPSC), catalyst recycle, living radical polymerization

## Abstract

Poly(ionic liquid)s (PILs) have become the frontier domains in separation science because of the special properties of ionic liquids as well as their corresponding polymers. Considering their function in separation, we designed and synthesized a thermoregulated PIL. That is, this kind of PIL could separate with an organic phase which dissolves the monomers at ambient temperature. When heated to the reaction temperature, they become a homogeneous phase, and they separate again when the temperature falls to the ambient temperature after polymerization. Based on this, a thermoregulated phase separated catalysis (TPSC) system for Cu-based atom transfer radical polymerization (ATRP) was constructed. The copper catalyst (CuBr_2_) used here is easily separated and recycled in situ just by changing the temperature in this system. Moreover, even when the catalyst had been recycled five times, the controllability over resultant polymers is still satisfying. Finally, only 1~2 ppm metal catalyst was left in the polymer solution phase, which indicates the really high recycling efficiency.

## 1. Introduction

Atom transfer radical polymerization (ATRP) is one of the most widely used radical polymerizations, and it is well-known for its numerous advantages, such as a wide range of suitable monomers, basically commercially available initiators, ligands, catalysts, and mild polymerization conditions. The resultant polymers can be synthesized with designed structures, including random, block, graft, star, gradient, hyper-branched (co) polymers, end functional polymers, etc. [1,2,3]. Usually, ATRP is mediated by transition metal catalysts in the presence of heat [4,5], light [6,7,8], electrochemistry [9,10,11,12], or microwave [13,14,15]. However, the transition metal residual in polymer product will pollute/color the polymers, which will not only result in the waste of metal catalysts, but also hinder ATRP industrialization and limit the application of polymer product especially in electronic and biomedical materials field.

In order to deal with the transition metal residual issue in ATRP, scientists developed a series of solutions to separate and recycle the transition metal catalysts [16]. Biphasic catalysis systems are effective ways. In solid–liquid biphasic catalysis systems, solid-supported catalysis by physical adsorption [17,18] and by covalent bonding [19,20,21,22], as well as immobilized/soluble hybrid [23,24] systems are easily removed by differences in physical properties between the solid phase and the liquid phase. However, these methods usually result in poor control over polymerization.

Especially, in liquid–liquid biphasic catalysis systems, the thermoregulated phase separation catalysis (TPSC) system, which was reported by Jin’s group in an organic reaction system [25], has unique advantages with the properties of “one phase reaction coupled with two-phase separation”. In the TPSC biphasic system, the metal catalysts and ligands are less soluble in nonpolar organic solvents at room temperature but dissolve in temperature-sensitive polar solvents. When heated above the critical temperature, the two phases are infinitely miscible to perform the reaction homogeneously, and the two phases are separated again when the reaction is cooled to room temperature. The temperature-sensitive phase containing the catalyst complex can be separated from the nonpolar organic phase containing production easily. Recently, we have extended the concept of TPSC to an ATRP process [26,27,28,29,30]. Herein, the ATRP catalyst complexes dissolve in one phase (usually in a polar solvent) while monomer and other substrates with excellent solubility in another phase (usually a nonpolar solvent). The two solvents dissolve unlimitedly and react in a homogeneous condition when heating the polymerization temperature; however, a thorough phase separation happens easily and spontaneously when cooling down to ambient or lower temperature, and the catalyst complexed can be separated from the polymer phase by an easy and efficient decantation. Due to the good combination of homogeneous and heterogeneous separation, this system ensures polymer control and prominence of catalyst selectivity while recycling the catalyst.

In traditional ATRP processes, a lower oxidation state transition metal (e.g., CuBr, FeCl_2_) was used as the original catalyst, which can be easily oxidized to a higher oxidation state during operation. Therefore, to obtain consistent results, special handling procedures are required, and the preformed catalysts must be stored under an inert atmosphere. Oxygen or other oxidants should be removed from the system prior to addition of the catalyst in the lower oxidation state. Therefore, the process of catalyst complex handling can be challenging [16]. In order to overcome the drawbacks of traditional ATRP, some improved ATRP methods, such as activators (re)generated by electron transfer (A(R)GET) ATRP [31,32,33], supplemental activators and reducing agents (SARA) ATRP [34,35], and initiators for continuous activator regeneration ATRP (ICAR ATRP) [36,37] have been reported. In these methods, higher oxidation state transition metals (e.g., CuBr_2_, FeCl_3_) are used, which facilitate the operation of catalyst separation and recycling. In the ICAR ATRP system, a thermal initiator (e.g., azobis(isobutyronitrile) (AIBN)) is used to generate radicals, which can react with higher oxidation state transition metals to generate active catalysts (lower oxidation state transition metal) in situ. Therefore, AIBN actually acts as a reducing agent in this case.

It is well known that ionic liquids (ILs) are salts that are liquids at low temperature (<100 °C) which present a new class of solvents [38]. They are famous for their plenty of unique strengths, such as low melting point, no measurable vapor pressure, highly thermostable, adjustable density and viscosity [39], satisfying coordination ability with organic/inorganic substances, easy to handle and get raw materials, high design-ability, etc. [40]. Poly(ionic liquid)s (PILs) refer to a kind of ionic polymers connected through a polymeric backbone to form a macromolecular architecture [30]. Therefore, it is a kind of a unique material which has the special qualities both of polymers and ionic liquids. PILs can be obtained just by the chain polymerizing of ionic liquids monomers [41]. Since Salamone et al. first polymerized several vinylimidazolium based ILs in the 1970s [42] various PILs based on different ionic liquid monomers have been polymerized. Most PILs gotten nowadays are solid with a relatively low glass transition temperature, which means that they are liquids at ambient temperature. Due to their charge features, PILs can stabilize catalytically active metal and metal oxide nanoparticles [43]. As a result, PIL-based polymerization systems as supports facilitate the recovery, recycling, and further use of the transition metal catalysts in comparison to molecular ILs [44,45]. Compared with ILs, the viscosities of PILs would not change greatly with the temperature changes, making it a stable phase in polymerization system [46,47,48,49,50].

**Scheme 1:**
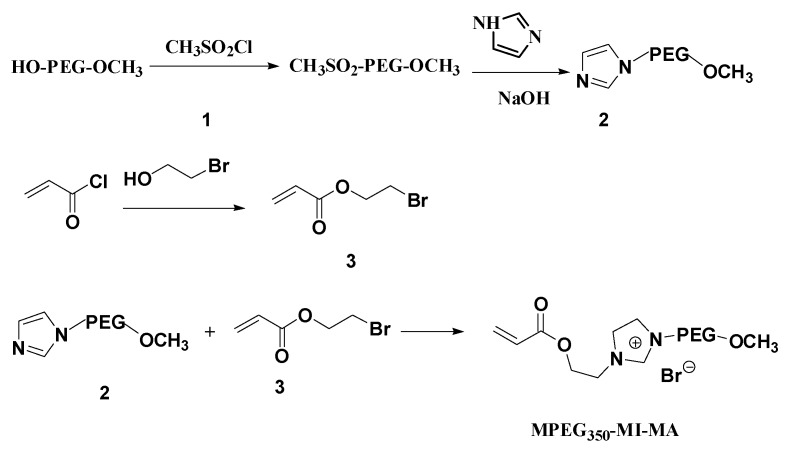
Synthetic pathway of the thermoregulated ionic liquid monomer MPEG_350_-MI-MA.

Combination of the advantages of TPSC, ICAR ATRP, and PILs, in this work, we designed a recycled thermoregulated PIL which are used in the ATRP system as a solvent and constructed a PIL/organic biphasic TPSC system for catalyst separation and recycling. Herein, CuBr_2_ was used as the original catalyst, tris ((2-pyridyl) methyl) amine (TPMA) as the ligand, ethyl-2-bromo-2-phenyl acetate (EBPA) as the ATRP initiator, and AIBN as the reducing agent. In addition, methyl methacrylate (MMA) is an important monomer in industry, which plays a vital role in manufacturing of organic glass, coatings, adhesives, and medical polymers [51], therefore MMA was used as a model monomer for this novel polymerization system. In addition, we chose a thermoregulated IL monomer, MPEG350-MI-MA, which was first synthesized in our group to polymerize the aim PIL [30]. The synthetic pathway is shown in Scheme 1. Furthermore, the resultant PIL and benzene were selected as thermoregulated phase and organic phase, respectively. In this novel system, a transition metal catalyst could be easily separated just by changing the polymerization temperature and this has nearly no negative effect on catalytic efficiency, polymer molecular weight, or molecular weight distribution.

## 2. Experimental Section

### 2.1. Materials

**Scheme 2:**
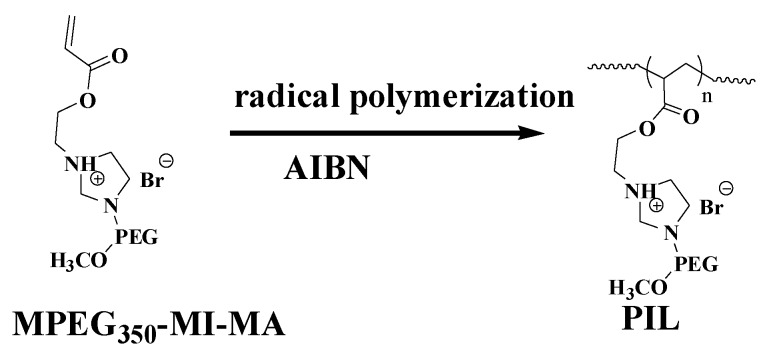
Synthetic pathway of poly(ionic liquid) (PIL).

Methyl methacrylate (MMA, +99%) purchased from Shanghai Chemical Reagents Co. Ltd. (Shanghai, China) were purified by passing through a neutral alumina column to remove inhibitors. Tris ((2-pyridyl) methyl) amine (TPMA, 98%, Sigma-Aldrich Co. Ltd., Shanghai, China) was used as received. 2,2′-Azobis (isobutyronitrile) (AIBN, 97%, Shanghai Chemical Reagents Co. Ltd.) was recrystallized from ethanol and dried at room temperature under vacuum before use. Monomethoxy poly(ethylene glycol)-350 (MPEG_350_, number-average molecular weight 350 g/mol, J&K Scientific, Shanghai, China), ethyl-2-bromo-2-phenyl acetate (EBPA, 97%, J&K), sodium triacetoxyborohydride (97%, Saan Chemical Technology Co. Ltd., Shanghai, China), acryloyl chloride (97%, Energy Chemical), and 2-bromoethanol (+96%, Shanghai Titan Technology Co., Ltd., Shanghai, China) were used as received. Copper bromide (CuBr_2_, analytical reagent), acetic acid glacial (analytical agent), tetraethylammonium bromide (+97%), triethylamine (N(Et)_3_, analytical reagent), methanesulfonyl chloride (analytical reagent), saturated sodium bicarbonate, benzene (analytical reagent), toluene (analytical agent), methanol (analytical agent), methylene dichloride (analytical agent), tetrahydrofuran (THF, analytical reagent), cyclohexane (analytical reagent), *n*-hexane (analytical reagent), and all other chemicals were obtained from Shanghai Chemical Reagents Co. Ltd. and used as received unless mentioned. The liquid ionic monomer, and thermoregulated poly (ionic liquid) were synthesized separately according to the procedures shown in Scheme 1 and Scheme 2.

### 2.2. Synthesis of the Thermoregulated Poly(Ionic Liquid) (PIL)

The synthetic pathway of the thermoregulated ionic liquid monomer MPEG_350_-MI-MA is shown in Scheme 1. Intermediates 1 and 2 were synthesized according to the reported literature [30]. Besides, the intermediate 3 was synthesized according to another previous work [52]. 15 mmol of MPEG_350_-MI-MA and 0.246 g AIBN (1.5 mmol) were added to a dried Schelenk tube with a stir bar, and then 10 mL of DMSO was also added to the tube. After bubbled with argon for 15 min, the tube was sealed and settled under 80 °C for three days. When the reaction was finished and the mixture was cooled to room temperature, washed the resulted solution with methanol for 3–5 times, then the poly (ionic liquid) (PIL) was obtained. The PIL was determined by gel permeation chromatograph (GPC) with *M*_n,GPC_ = 26,300 g/mol and *M*_w_/*M*_n_ = 1.46.

### 2.3. Typical Procedure for the TPSC-Based ICAR ATRP of MMA and Catalyst Recycling

The typical procedure for the TPSC-based ICAR ATRP of MMA is demonstrated below with a molar ratio of [MMA]_0_/[EBPA]_0_/[CuBr_2_]_0_/[TPMA]_0_/[AIBN]_0_ = 200:1:1:3:1. CuBr_2_ (10.4 mg, 0.047 mmol), EBPA (8.3 µL, 0.047 mmol), MMA (1 mL, 9.4 mmol), PIL (200 mg, 0.108 mmol), AIBN (7.8 mg, 0.047 mmol), and benzene (3.0 mL) were added into a 5 mL ampoule with a clean stir bar. The ampoule was bubbled with argon for about 15 min to eliminate the dissolved oxygen, and then flame-sealed. The sealed ampoule was heated to 70 °C for polymerization. After the desired time of polymerization, the ampoule was taken out and the ampoule was cooled by immersing it into iced water. After the system was completely phase-separated, the ampoule was broken, and transferred the upper polymer solution into THF to get a diluted one, then the mixture was precipitated in methanol. The precipitated product was filtered off with suction from a vacuum distillation flask. To obtain a dried product, the filtered product was placed in a vacuum oven at 30 °C for about 3–4 h. The weight of the dried product was measured and the monomer conversion was calculated according to it. Finally, The PIL phase containing the catalyst was transferred to a new ampoule and fresh EBPA (8.3 µL, 0.047 mmol), MMA (1 mL, 9.4 mmol), AIBN (7.8 mg, 0.047 mmol) and benzene (3 mL) were also added for catalyst recycling experiments. All remaining polymerization operations were the same as above.

### 2.4. Typical Procedure for Chain Extension of PMMA

A typical chain extension polymerization procedure of PMMA with the molar ratio of [MMA]_0_/[PMMA]_0_/[CuBr_2_]_0_/[TPMA]_0_/[AIBN]_0_ = 200:1:1:3:1 was as follows: The polymer obtained was filtered with neutral alumina and reprecipitated, then it was dried in a vacuum oven to a constant weight. A predetermined quantity of PMMA (0.024 mmol) was added in a dry 5 mL ampule with a stir bar as the macro-initiator, then the corresponding amount of CuBr_2_ (0.047 mmol), PIL (0.108 mmol), MMA (9.4 mmol), AIBN (0.047 mmol), and benzene (3.0 mL) were added. The rest of the procedure was the same as the typical procedure for the TPSC-based ICAR ATRP described above.

### 2.5. Characterizations

The number-average molecular weight (*M*_n,GPC_) and molecular weight distribution (*M*_w_/*M*_n_) values of PMMA were determined using a TOSOH HLC-8320 gel permeation chromatograph (GPC) equipped with a refractive-index detector (TOSOH), using TSKgel guardcolumn SuperMP-N (4.6 × 20 mm) and two TSKgel SupermultiporeHZ-N (4.6 × 150 mm) with measurable molecular weights ranging from 10^3^ to 1 × 10^6^ g·mol^−1^. THF was employed as the eluent at a flow rate of 0.35 mL·min^−1^ at 40 °C. The GPC samples were injected using a TOSOH plus auto-sampler and calibrated with PMMA standards purchased from TOSOH. ^1^H NMR spectra were recorded on an INOVA 400 MHz nuclear magnetic resonance (NMR) instrument using CDCl_3_ and DMSO-*d*_6_ as the solvents and tetramethylsilane (TMS) as an internal standard at ambient temperature.

## 3. Results and Discussion

### 3.1. Selection of Solvent for TPSC-Based ICAR (Initiators for Continuous Activator Regeneration) ATRP System

**Figure 1:**
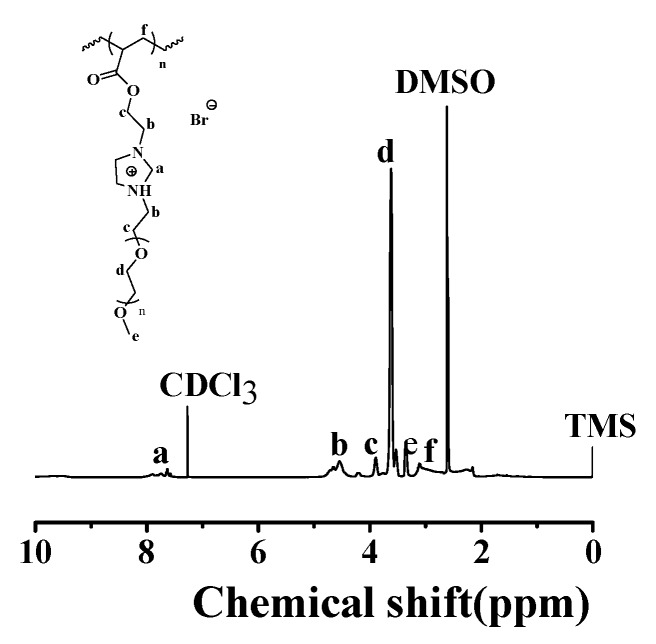
^1^H NMR spectrum of poly(ionic liquid) (PIL) with CDCl_3_ and DMSO-*d*_6_ as mixed solvents and tetramethylsilane (TMS) as an internal standard.

**Figure 2:**
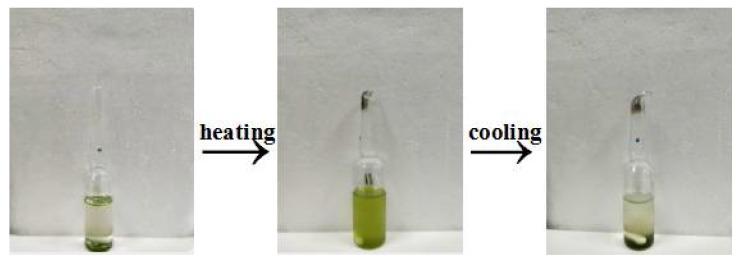
Photographs of thermoregulated phase separated catalysis (TPSC)-based ICAR-ATRP (initiators for continuous activator regeneration-atom transfer radical polymerization) in PIL/benzene biphasic solvent system.

The PIL (*M*_n,GPC_ = 26,300 g/mol, *M*_w_/*M*_n_ =1.46) was confirmed with 1H NMR spectroscopy. From Figure 1, it can be seen that the monomer’s double bond disappeared at δ = 5.84–6.47 ppm and a new group of chemical shifts were found at δ = 2.65–2.95 ppm (f in Figure 1) assigned to polymer backbone hydrogen bonds, which indicated that the PIL was obtained successfully. In order to construct the TPSC-based ATRP system, we screened out the optimal solvent system firstly. The results are shown in Table 1. Six organic solvents including p-xylene, o-xylene, toluene, benzene, cyclohexane, and n-hexane were investigated to form different mixed solvents with the synthesized PIL. At room temperature, only cyclohexane/PIL solvent pair was miscible, which indicating that it did not meet the biphasic solvent requirement of TPSC system. After increasing the temperature to 70–90 °C, it is found that only benzene/PIL solvent pair became miscible completely, namely forming a homogeneous solution. Therefore, benzene was selected as the optimal organic solvent applied in this TPSC system. Actually, as shown in Figure 2, the homogeneous polymerization and heterogeneous separation process could be easily realized just by changing the reaction temperature with benzene/PIL solvent system.

Subsequently, we investigated the effect of types of ligands (Me6TRAN, PMDETA, TPMA, and TDA-1), reducing agents (AsAc, AsAc-Na, glucose, and AIBN) on polymerization. As listed in Table 2, all the polymerizations could be performed smoothly (Entries 1–8, Table 2). However, TPMA and AIBN were selected as the ligand and reducing agent, respectively, by considering polymerization rate and controllability over PMMA molecular weight and its distribution. Therefore, an optimal ICAR ATRP system could be constructed by using MMA as the model monomer, EBPA as the ATRP initiator, CuBr_2_ as the catalyst, TPMA as the ligand, AIBN as the reducing agent, and benzene/PIL as the solvent pair system. In addition, we also investigated the effect of molar ratio of CuBr_2_ to AIBN, and the results are shown in Table 3. It can be seen from Table 3 that the polymerization could be successfully carried out with a wide range of [AIBN]_0_:[CuBr_2_]_0_ ([AIBN]_0_:[CuBr_2_]_0_ = 0.5–2:1). However, the polymerization rate increased with the increase of the amount of AIBN since more active catalyst CuBr could be generated correspondingly as expected by ICAR ATRP mechanism [37].

### 3.2. Polymerization Kinetics of MMA

**Figure 3:**
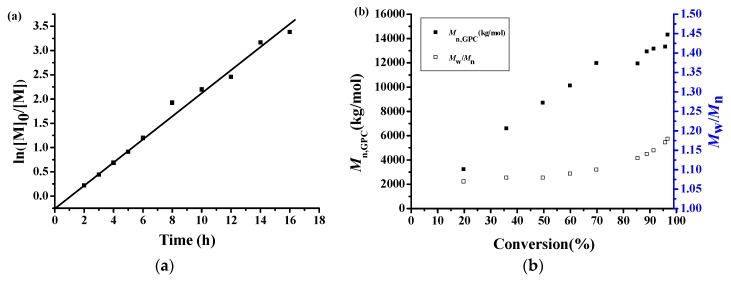
In([M]_0_/[M]) as a function of (**a**) time and (**b**) number-average molecular weight (*M*_n,GPC_) and molecular weight distribution (*M*_w_/*M*_n_) versus monomer conversion for TPSC-based ICAR ATRP of MMA. [MMA]_0_/[EBPA]_0_/[CuBr_2_]_0_/[TPMA]_0_/[AIBN]_0_ = 200:1:1:3:0.8, *V*_MMA_ = 1.0 mL, *V*_benzene_ = 3.0 mL, *m*_PIL_ = 200 mg and, temperature = 70 °C.

To make a relatively low viscosity reaction condition and a fit polymerization rate as well as the better controllability of polymerization, we finally chose the molar ratio of [MMA]_0_/[EBPA]_0_/[CuBr2]_0_/[TPMA]_0_/[AIBN]_0_ = 200:1:1:3:0.8 and 3.0 mL of benzene, 200 mg PIL to further investigate the polymerization kinetics of MMA in this polymerization system. The first-order kinetics (Figure 3a) indicated that the propagating radicals in the system remained constant throughout the polymerization up to high monomer conversion (more than 95%). Figure 3b shows that *M*_n,GPC_ increased linearly with monomer conversion and the molecular weight distributions kept narrow (*M*_w_/*M*_n_ < 1.20). These results indicated the “living”/controlled features of the TPSC-based ICAR ATRP of MMA in PILL/benzene biphasic system.

### 3.3. Chain-End Analysis and Chain Extension

**Figure 4:**
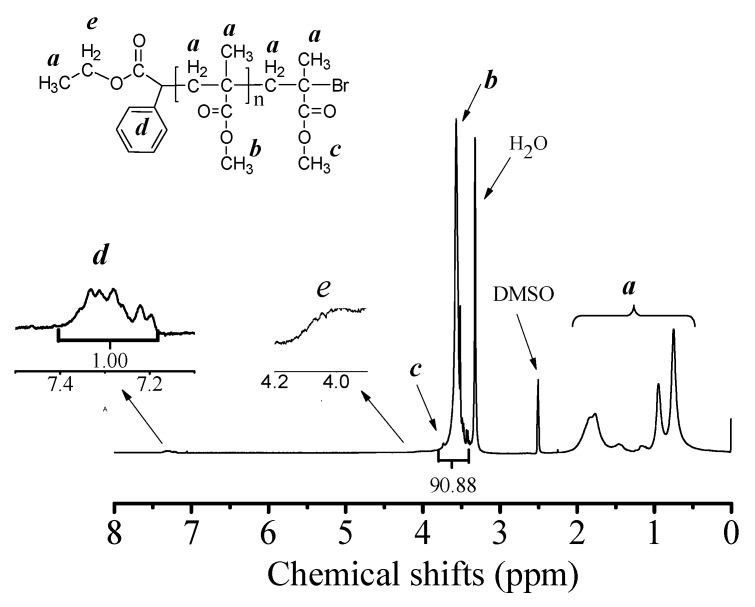
^1^H NMR spectrum of the resultant PMMA (*M*_n,GPC_ = 4250 g/mol, *M*_w_/*M*_n_ = 1.22) via TPSC-based ICAR ATRP of MMA using DMSO-*d*_6_ as the solvent and TMS as the internal standard.

**Figure 5:**
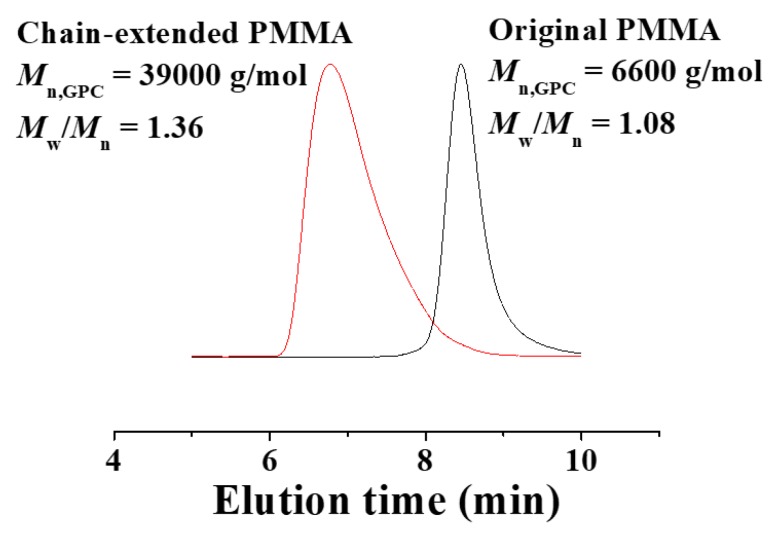
Gel permeation chromatograph (GPC) traces of before and after chain extension using PMMA (obtained by TPSC-based ICAR ATRP of MMA) as the macroinitiator. Original PMMA:[MMA]_0_:[EBPA]_0_:[CuBr_2_]_0_:[TPMA]_0_:[AIBN]_0_ = 200:1:1:1:0.8, *V*_MMA_ = 1.0 mL, *V*_benzene_ = 3.0 mL and *m*_PIL_ = 200.0 mg, temperature = 70 °C. Chain-extended PMMA:[MMA]_0_:[PMMA]_0_:[CuBr_2_]_0_:[TPMA]_0_: [AIBN]_0_ = 200:0.05:1:1:0.8, *V*_MMA_ = 1.0 mL, *V*_benzene_ = 3.0 mL and *m*_PIL_ = 200 mg, temperature = 70 °C.

In order to verify the structure of the resultant polymer and the chain-end functionality, we made analysis of a resultant PMMA (*M*_n,GPC_ = 4250 g/mol, *M*_w_/*M*_n_ = 1.22) by ^1^H NMR spectroscopy. From the ^1^H NMR spectrum of the polymer (Figure 4), it can be seen that the chemical shifts at δ = 4.0–4.1 ppm (e in Figure 4) and δ = 7.15–7.35 ppm (as in Figure 4) are attributed to the methyl of initiator EBPA and hydrogen of aromatic rings, respectively. This indicated that the initiator EBPA moieties had successfully attached on the structure of the polymers. The chemical shifts at δ = 3.78 ppm (c in Figure 4) are attributed to the bromine-terminated methyl ester group at the chain end [13]. In addition, to further demonstrate the “living” feature of the resulting polymers, we used the resultant PMMA as a macroinitiator for the chain extension via TPSC-based ICAR ATRP method. The molecular weight increased to *M*_n,GPC_ = 39,000 g/mol from *M*_n,GPC_ = 6600 g/mol after chain extension, while the molecular weight distribution kept relatively narrow (*M*_w_/*M*_n_ = 1.36) (Figure 5). The successful chain extension further demonstrated the “living” character of this novel ATRP catalyst separation and recycling system.

### 3.4. Catalyst Recycling and Reuse

**Figure 6:**
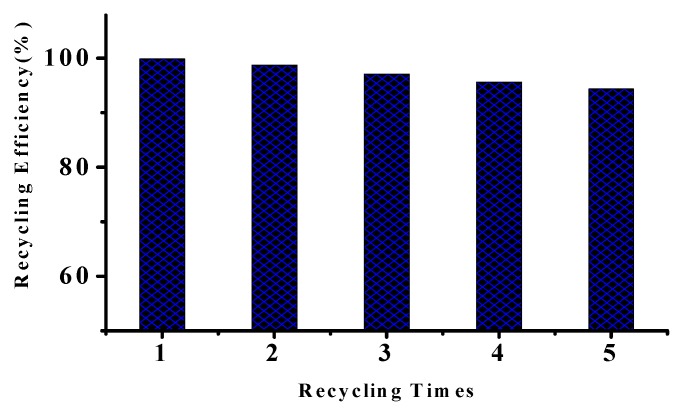
Recycling efficiency as a function of recycling times. Polymerization conditions: [MMA]_0_/[EBPA]_0_/[CuBr_2_]_0_/[TPMA]_0_/[AIBN]_0_ = 200:1:1:3:1, *V*_MMA_ = 1.0 mL, *V*_benzene_ = 3.0 mL, *m*_PIL_ = 200 mg, *t* = 4.5 h, and temperature = 70 °C. Recycling efficiency was calculated by the percentage of residual copper catalyst in poly(ionic liquid) phase to the original copper catalyst, which was determined by ICP.

In the constructed TPSC-based ICAR ATRP, the recycling efficiency is the most important parameter. Therefore, we conducted the following experiments using the recovered PIL phase. The monomer (MMA), ligand (TPMA), reducing agent (AIBN), and organic solvent (benzene) needed for the typical MMA polymerization were respectively added to the recovered PIL phase and carried out the subsequent recovered polymerization. When the polymerization was completed, a part of the polymer phase was taken out to have an inductively coupled plasma (ICP) test to determine the amount of transition metal remaining in the polymer solution. The recycling experiments and corresponding results are shown in Table 4. It can be seen that after five recovery experiments, the catalysis efficiency of the catalyst was still maintained at a high level, and the resulting polymers kept narrow molecular weight distributions. Importantly, the residual Cu catalyst in polymer solution phase was less than 2.2 ppm in every recycling experiment. That is to say even after five recycling polymerizations the catalyst recycling efficiency still remained high (more than 94%, as is shown in Figure 6). These results was much better than that (less than 90%) reported by our previous similar TPSC-based ICAR ATRP in *p*-xylene/PEG-200 biphasic system [27].

## 4. Conclusions

A novel TPSC-based ICAR ATRP system with high catalyst recycling efficiency was successfully constructed via a thermoregulated PIL/benzene as the solvent pair strategy. In this system, the Cu catalyst is miscible with the monomer/polymer at the polymerization temperature (70 °C); when cooled to room temperature, the ATRP catalyst dissolved in PIL was in situ separated from the polymer organic solution (benzene solution) easily and recycled for the next polymerization facilely. Therefore, this strategy can avoid the conventional tedious post-treatment steps of recycling catalyst, which will be much beneficial for the industrial process of ATRP.

## Figures and Tables

**Table 1:** Selection of organic solvents with PIL (174 mg) for the TPSC-based polymerization ^a^.

Solvent (2.0 mL)	25 °C	70–90 °C	25 °C
*p*-Xylene	I	S	I
*o*-Xylene	I	S	I
Toluene	I	S	I
Benzene	I	M	I
Cyclohexane	M	M	M
*n*-Hexane	I	S	I

^a^ **I** indicates that the two solvents are immiscible at investigated temperature; **S** indicates that the two solvents are slightly miscible at investigated temperature; and **M** indicates that the two solvents are miscible at investigated temperature.

**Table 2:** Effect of kinds of ligand and reducing agent on polymerization.

Entry	Ligand	Reducing Agent	Time (h)	Conv. (%)	*M*_n,th_^a^ (g/mol)	*M*_n,GPC_ (g/mol)	*M*_w_/*M*_n_
1	TPMA	AIBN	11.5	73.1	14,800	14,600	1.17
2	PMDETA	AIBN	10	81.9	16,400	10,300	1.39
3	Me6TRAN	AIBN	10	83.9	16,800	8700	1.51
4	TDA-1	AIBN	10	72.5	14,500	29,100	1.58
5	TPMA	AIBN	5.5	69.9	14,000	12,000	1.31
6	TPMA	Glucose	57	46.4	9500	11,400	1.11
7	TPMA	AsAc	57	62.5	12,700	16,200	1.11
8	TPMA	AsAc-Na	5.5	66.1	13,400	19,300	1.12

Polymerization conditions: [MMA]_0_:[EBPA]_0_:[CuBr_2_]_0_:[ligand]_0_:[reducing agent]_0_ = 200:1:1:3:1, *V*_MMA_ = 1.0 mL, *V*_benzene_ = 3.0 mL and *m*_PIL_ = 200 mg, temperature = 70 °C. ^a^ *M*_n,th_ = ([M]_0_/[EBPA]_0_) × *M*_w,MMA_ × conv. %. TPMA: tris(2-pyridylmethyl)amine; PMDETA: pentamethyldiethylenetriamine; Me_6_TRAN: tris(2-dimethylaminoethyl)amine; TDA-1: tris(3,6-dixa-heptyl)amine; AsAc: ascorbic acid; AsAc-Na: sodium ascorbate.

**Table 3:** Effect of molar ratio of [CuBr_2_]_0_:[AIBN]_0_ on polymerization.

Entry	y/x	Time (h)	Conv. (%)	*M*_n,th_^a^ (g/mol)	*M*_n,GPC_ (g/mol)	*M*_w_/*M*_n_
1	0.5	5.5	19.8	4000	5600	1.10
2	0.8	5.5	30.2	6000	8100	1.11
3	1.0	5.5	39.4	7900	8900	1.11
4	1.5	5.5	43.6	8700	10,200	1.13
5	2.0	5.5	46.3	9300	10,300	1.14

Polymerization conditions: [MMA]_0_:[EBPA]_0_:[CuBr_2_]_0_:[TPMA]_0_:[AIBN]_0_ = 200:1:x:3:y, *V*_MMA_ = 1.0 mL, *V*_benzene_ = 3.0 mL and m_PIL_ = 200 mg, temperature = 70 °C. ^a^ *M*_n,th_ = ([M]_0_/[EBPA]_0_) × *M*_w,MMA_ × conv. %.

**Table 4:** Polymerization results of PIL recycling via TPSC-based ICAR ATRP ^a^*.*

Entry	Recycling Times	Conv. (%)	*M*_n,th_^b^ (g/mol)	*M*_n,GPC_ (g/mol)	*M*_w_/*M*_n_	CR ^c^ (ppm)
1	1	59.8	11,980	18,500	1.12	1.3
2	2	55.7	11,200	15,600	1.09	1.5
3	3	60.2	12,100	18,100	1.12	2.2
4	4	52.4	10,500	18,900	1.10	2.0
5	5	52.3	13,000	12,200	1.07	1.7

^a^ Polymerization conditions: [MMA]_0_:[EBPA]_0_:[CuBr_2_]_0_:[TPMA]_0_:[AIBN]_0_ = 200:1:1:3:0.8, *V*_MMA_ = 1.0 mL, *V*_benzene_ = 3.0 mL, *m*_PIL_ = 200 mg for the first run, *t* = 4.5 h, and temperature = 70 °C; ^b^ *M*_n,th_ = ([M]_0_/[initiator]_0_) × *M*_w,MMA_ × conv.%; ^c^ CR means the catalyst residual in polymer solution phase (benzene phase), which was determined by inductively coupled plasma (ICP).
